# Efficient simulation of stochastic chemical kinetics with the Stochastic Bulirsch-Stoer extrapolation method

**DOI:** 10.1186/1752-0509-8-71

**Published:** 2014-06-18

**Authors:** Tamás Székely, Kevin Burrage, Konstantinos C Zygalakis, Manuel Barrio

**Affiliations:** 1 Department of Computer Science, University of Oxford, Oxford, OX1 3QD, UK; 2 Department of Mathematics, Queensland University of Technology, Brisbane, Qld 4001, Australia; 3 Mathematical Sciences, University of Southampton, Southampton, SO17 1BJ, UK; 4 Departamento de Informática, Universidad de Valladolid, 47011 Valladolid, Spain

**Keywords:** Stochastic simulation, Discrete stochastic methods, Bulirsch-Stoer, *τ*-leap, High-order methods

## Abstract

**Background:**

Biochemical systems with relatively low numbers of components must be simulated stochastically in order to capture their inherent noise. Although there has recently been considerable work on discrete stochastic solvers, there is still a need for numerical methods that are both fast and accurate. The Bulirsch-Stoer method is an established method for solving ordinary differential equations that possesses both of these qualities.

**Results:**

In this paper, we present the Stochastic Bulirsch-Stoer method, a new numerical method for simulating discrete chemical reaction systems, inspired by its deterministic counterpart. It is able to achieve an excellent efficiency due to the fact that it is based on an approach with high deterministic order, allowing for larger stepsizes and leading to fast simulations. We compare it to the Euler *τ*-leap, as well as two more recent *τ*-leap methods, on a number of example problems, and find that as well as being very accurate, our method is the most robust, in terms of efficiency, of all the methods considered in this paper. The problems it is most suited for are those with increased populations that would be too slow to simulate using Gillespie’s stochastic simulation algorithm. For such problems, it is likely to achieve higher weak order in the moments.

**Conclusions:**

The Stochastic Bulirsch-Stoer method is a novel stochastic solver that can be used for fast and accurate simulations. Crucially, compared to other similar methods, it better retains its high accuracy when the timesteps are increased. Thus the Stochastic Bulirsch-Stoer method is both computationally efficient and robust. These are key properties for any stochastic numerical method, as they must typically run many thousands of simulations.

## Background

Microscopic processes with few interacting components can have considerable effects at the macroscopic scale [1-3]. Stochasticity is a defining property of these processes, which can have so few component particles that random fluctuations dominate their behaviour [4,5]. Stochastic simulation methods take proper account of these fluctuations, as opposed to deterministic methods that assume a system does not deviate from its mean behaviour [6]; although deterministic methods can often be useful for an approximate description of the dynamics of a system, their results are not always representative [7,8].

A common stochastic modelling approach is to consider the system as a continuous-time Markov jump process [9]. The stochastic simulation algorithm (SSA) of Gillespie [10] is a simple and exact method for generating Markov paths. However, because it keeps track of each reaction, it can be too computationally costly for more complex systems or those with frequent reactions. Many approximate methods have since been developed, which use similar principles as the SSA but group many reactions into a single calculation, reducing computational time (for a recent review, see [11]).

The first of these is commonly called the Euler or Poisson *τ*-leap [12]; it corresponds to the Euler method for ordinary differential equations (ODEs), and samples a Poisson random variable at each step. The original stepsize selection procedure has since been modified to improve accuracy [13,14]. To deal with issues of negative populations, Tian and Burrage [15] and Chatterjee *et al.*[16] introduced the binomial *τ*-leap, which samples a binomial random variable at each step. In addition, a newer binomial *τ*-leap [17] and a multinomial *τ*-leap [18] have since been proposed.

In his seminal paper, Gillespie also proposed the midpoint *τ*-leap, a higher-order method that allows for larger timesteps by reducing the inherent bias of the *τ*-leap [12]. More recently, other higher-order methods have been developed, such as the unbiased *τ*-leap [19], random-corrected *τ*-leap [20], *θ*-trapezoidal *τ*-leap [21], and extrapolated *τ*-leap [22]. Rather than focussing on adaptively optimising the timestep to reduce processing time, these methods instead improve their order of accuracy so that they find more accurate results for a given stepsize. Because of this, they can use larger timesteps for a desired error level, reducing processing time.

In this paper, we introduce a new adaptive-stepsize method for simulating discrete Markov paths, which we call the Stochastic Bulirsh-Stoer (SBS) method. This is inspired by the deterministic method of the same name, a very accurate method for solving ODEs, based on Richardson extrapolation. Its high accuracy due to extrapolation, and its ability to adaptively maximise the timestep make the Bulirsch-Stoer method one of the most powerful ODE solvers. Possessing these same advantages, our SBS method is a very efficient and accurate new discrete stochastic numerical method.

The SBS calculates several approximations for the expected number of reactions occurring per timestep *τ* using stepsizes *τ*/2, *τ*/4, and so on, and extrapolates these to arrive at a very accurate estimate; the state of the system is then found by sampling a Poisson distribution with this parameter. The SBS is in some ways similar to the extrapolated *τ*-leap methods we proposed in a previous paper [22]. These involve running simulations with a *τ*-leap method of choice over the full time period of interest, and then taking moments and extrapolating them. The SBS is also based on extrapolation, but the extrapolation is carried out *inside* each timestep, rather than at the end of the simulation, allowing *τ* to be optimised at each step.

### Overview of stochastic methods

We start with a chemical system of *N* species and *M* reactions, interacting in a fixed volume *Ω* at constant temperature, that is both well-stirred and homogeneous. Thus we assume that individual molecules undergo both reactive collisions and non-reactive ones, and the latter is more frequent than the former, mixing the molecules thoroughly [11]. Individual molecules are not tracked, rather it is their total numbers that we are interested in. These are stored in an *N*×1 state vector, **x**≡**X**(*t*)≡(*X*_1_,…,*X*_*N*_)^*T*^, that contains the integer number of each type of molecule at some time *t*. Reactions are represented by an *N*×*M* matrix consisting of stoichiometric vectors ***ν***_*j*_≡(*ν*_1*j*_,…,*ν*_*N* *j*_)^*T*^,*j*=1,…,*M*, which dictate how each reaction changes the system state, and an *M*×1 vector of propensity functions *a*_*j*_(**x**), where *a*_*j*_(**x**)*dt* gives the probabilities of each reaction occurring in an infinitesimal time interval *dt*. Together, these three variables fully characterise the chemical system as it evolves through time. In this paper, we adopt the following notation: a bold font variable refers to an *N*×1 vector, e.g. **X**(*t*), and unless otherwise specified the indices *i*=1,…,*N* and *j*=1,…,*M*.

A conceptually simple way of simulating problems using this framework is the SSA of Gillespie [10]. It steps along reaction-by-reaction, at each step calculating the (exponentially-distributed) time until the next reaction *τ*, and the reaction *j*^′^ that will occur. The state vector is evolved in time according to the update equation

(1) $$X_{n+1}=X_{n}+\overset{M}{\underset{j=1}{\sum }}\nu_{j}K_{j},\;K_{j}=\{\begin{array}{c} 1\;\text{if}\;\;j=j^{\prime },\\ 0\;\text{otherwise,} \end{array}$$

i.e. only one reaction occurs over [*t*,*t*+*τ*). Both *τ* and *j*^′^ are sampled randomly as required by the stochastic nature of the process: 

The SSA is a *statistically exact* method for generating Monte Carlo paths. That is, a histogram built up from an infinite number of simulations of the SSA will be identical to the true histogram of the system. It is the stochastic method of choice for many researchers, but it has one main limitation: as with other stochastic methods, many realisations (usually starting at 10^4^ or 10^5^) must be simulated to get a reasonable idea of the histogram shape, and SSA simulations can be slow depending on the problem.

The *τ*-leap [12] was introduced by Gillespie as a faster alternative to the SSA. It improves speed by evaluating many reactions in one step, which is typically much larger than that of the SSA. This allows the *τ*-leap to be generally very fast compared to the SSA, but also means that it is not exact. Assuming *τ* is sufficiently small so that the propensities do not change significantly during each step (the ‘leap condition’), the number of reactions occurring during [*t*,*t*+*τ*), *K*_*j*_, is a Poisson random variable [11,12] with parameter *a*_*j*_(**x**)*τ*. The simplest *τ*-leap implementation is the Euler *τ*-leap with fixed stepsize. 

This method is effective but a little crude: unless simplicity is the most important consideration or *τ* must be fixed, as is the case when used in the context of Richardson extrapolation [22], it is advisable to use more advanced *τ*-leap methods.

Adaptively changing *τ* at each step can give even greater gains in speed, and this is easily introduced into Algorithm 2. A successful approach in current implementations is to find *τ* such that the mean and variance of the *change in propensities over* [*t*,*t*+*τ*) are bounded by some fraction *ε*≪1 of *a*_*j*_(**X**_*n*_). The advantage of this is that *τ* is controlled to stick more closely to the leap condition, ensuring better accuracy, while at the same time maximimising *τ* for faster simulations. There have been several successive improvements for best selecting *τ*[12-14], and these methods can achieve a very high efficiency.

## Methods

### Extrapolation

Richardson extrapolation is a technique for increasing the order of accuracy of a numerical method by eliminating the leading error term(s) in its error expansion [23,24]. It involves numerically solving some deterministic function **Y**(*t*) at a given time *T*=*nτ* using the same solver with different stepsizes, where we define $Y_{T}^{\tau }$ as an approximation to **Y**(*T*) at time *T* using stepsize *τ*. **Y**(*T*) can be written as

$$Y(T)=Y_{T}^{\tau }+\varepsilon_{g}(\tau ),$$

where *ε*_*g*_(*τ*) is the error of the approximate solution compared to the true one. For a general numerical solver, *ε*_*g*_(*τ*) can be written in terms of powers of the stepsize *τ*:

(2) $$\varepsilon_{g}(\tau )=e_{k_{1}}\tau^{k_{1}}+e_{k_{2}}\tau^{k_{2}}+e_{k_{3}}\tau^{k_{3}}+\ldots ,$$

where the *e*_*k*_ are constant vectors and depend only on the final time and *k*_1_<*k*_2_<*k*_3_,…. Eq. (2) tells us that this method has order of accuracy *k*_1_.

**Figure 1:**
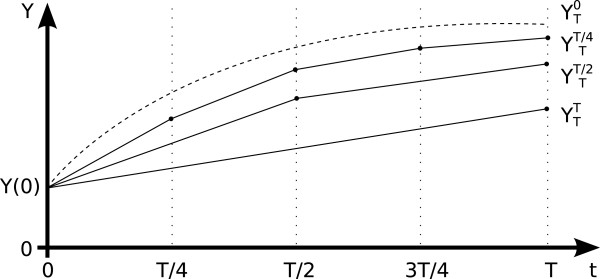
**Richardson extrapolation principle.** Three numerical solutions, with stepsizes $\tau_{1}=T,\tau_{2}=\frac{T}{2},\tau_{3}=\frac{T}{4}$ find estimates closer and closer to the true solution $Y(T)=Y_{T}^{0}$, i.e. the numerical solution in the limit of zero stepsize. They can be extrapolated to find an estimate very close to $Y_{T}^{0}$.

Essentially, Richardson extrapolation employs polynomial extrapolation of approximations $Y_{T}^{\tau_{q}},q=1,2,\ldots$ and *τ*_1_>*τ*_2_>…, to estimate $Y_{T}^{0}$, i.e. the numerical solution in the limit of zero stepsize, which corresponds to **Y**(*T*) (Figure 1). Each successive extrapolation removes the next leading error term, which is the largest contribution to the error, thereby increasing the accuracy of the numerical solution and allowing it to better estimate **Y**(*T*).

To demonstrate this, assume a numerical method with stepsize *τ* has an error expansion of

$$Y(T)-Y_{T}^{\tau }=e_{1}\tau +e_{2}\tau^{2}+O(\tau^{3})$$

For instance, the well-known Euler method for solving ODEs has such an error expansion. Now instead of *τ*, if we use a stepsize *τ*/2, the error expansion is

(3) $$Y(T)-Y_{T}^{\tau /2}=e_{1}\frac{\tau }{2}+e_{2}\frac{\tau^{2}}{4}+O(\tau^{3})$$

We can take $Y_{T}^{\tau ,\tau /2}=2Y_{T}^{\tau /2}-Y_{T}^{\tau }$, giving

(4) $$Y(T)-Y_{T}^{\tau ,\tau /2}=-e_{2}\frac{\tau^{2}}{2}+O(\tau^{3})$$

The leading error term has been removed, resulting in a higher-order approximation. This can be repeated to obtain an even higher order of accuracy by using more initial approximations $Y_{T}^{\tau_{1}},\ldots Y_{T}^{\tau_{q}}$, where *q* can be any integer and *τ*_1_>*τ*_2_>… *τ*_*q*_. We define $Y_{T}^{\tau_{1},\tau_{q}}$ as the extrapolated solution using initial approximations $Y_{T}^{\tau_{1}},\ldots Y_{T}^{\tau_{q}}$. The easiest way of visualising this is to build up a Neville table (also called a Romberg table) from the initial approximations (Table 1).

**Table 1:** **Neville table built from *q* initial approximations *YTτ***_**1**_**,…,*YTτ***_***q***_** with order *k***_**1**_** (first column) and extrapolated to find a solution of order *k***_***q***_**, that is**$Y_{T}^{\tau_{1},\tau_{q}}$

**Order**	***k***_**1**_	***k***_**2**_	***k***_**3**_	**…*k***_***q***_
	$Y_{T}^{\tau_{1}}$			
		$Y_{T}^{\tau_{1},\tau_{2}}$		
Approximate	$Y_{T}^{\tau_{2}}$		$Y_{T}^{\tau_{1},\tau_{3}}$	
solutions		$Y_{T}^{\tau_{2},\tau_{3}}$	⋮	$\ldots \;Y_{T}^{\tau_{1},\tau_{q}}$
	$Y_{T}^{\tau_{3}}$	⋮	$Y_{T}^{\tau_{q-2},\tau_{q}}$	
	⋮	$Y_{T}^{\tau_{q-1},\tau_{q}}$		
	$Y_{T}^{\tau_{q}}$			

The first column of the table contains the initial numerical approximations. These are then extrapolated to find the next column, and so on. For instance, with three initial solutions $Y_{T}^{\tau },Y_{T}^{\tau /2},Y_{T}^{\tau /4}$, then $Y_{T}^{\tau ,\tau /4}=\frac{4}{3}Y_{T}^{\tau /2,\tau /4}-\frac{1}{3}Y_{T}^{\tau ,\tau /2}$ (this is easily calculated by first writing down a similar formula to Eq. (3) for $Y_{T}^{\tau /4}$, then one similar to Eq. (4) for $Y_{T}^{\tau /2,\tau /4}$, and once more for $Y_{T}^{\tau ,\tau /4}$). At each subsequent column, the next leading error term is cancelled, giving a yet higher-order solution. The correct coefficients to calculate each new term of the Neville table can be found from

$$Y_{T}^{\tau_{q-r},\tau_{q}}=\frac{p^{k_{q}}\;Y_{T}^{\tau_{q-r+1},\tau_{q}}-Y_{T}^{\tau_{q-r},\tau_{q-1}}}{p^{k_{q}}-1},$$

where *p*=*τ*_*q*−*r*_/*τ*_*q*−*r*+1_ and *k*_*q*_ is the order of the solution at column *q* (c.f. Eq. (2)), and *r*=1,…,*q*−1. This can be generalised to *any* order method with *any* appropriate error expansion. The only condition for extrapolation is existence of an error expansion of the form in Eq. (2).

### Bulirsch-Stoer method

The Bulirsch-Stoer method is an accurate ODE solver based on Richardson extrapolation [25,26]. A Neville table is built by repeated extrapolation of a set of initial approximations with stepsizes that are different subintervals of a larger overall step *τ*, and is then used to find a very accurate solution. This happens *inside each timestep*, allowing *τ* to be varied between steps. A modified midpoint method (MMP, Algorithm 3) is used to generate the initial approximations in the first column of the table. This lends itself well to an extrapolation framework, as the MMP subdivides each step *τ* into $\hat{n}$ substeps $\hat{\tau }=\tau /\hat{n}$. Furthermore, crucially, the error expansion of the MMP contains only even powers of $\hat{\tau }$, resulting in fast convergence [27]. 

We give a brief overview of the deterministic Bulirsch-Stoer method here; Ref. [28] has an excellent description of the algorithm, as well as a guide to its implementation. At each step, a column of the Neville table, *k*, in which we expect the approximate solutions to have converged, as well as a stepsize *τ* are selected. The Neville table is then built up by running *k* MMPs, with stepsizes ${\hat{\tau }}_{1}=\tau /2,\ldots ,{\hat{\tau }}_{q}=\tau /n_{q}$, where *n*_*q*_=2*q*,*q*=1,2,…,*k* and successively extrapolating the appropriate numerical approximations. The convergence of the solutions is evaluated based on the internal consistency of the Neville table, that is, the difference between the most accurate solution in column *k* and that in column *k*−1: from Table 1, this is $\Delta Y(k,k-1)=Y_{\tau }^{{\hat{\tau }}_{1},{\hat{\tau }}_{k}}-Y_{\tau }^{{\hat{\tau }}_{2},{\hat{\tau }}_{k}}$. As successive initial approximations $Y_{\tau }^{{\hat{\tau }}_{q}}$ are added to the first column, the extrapolated results in each new column converge to the true solution and *Δ***Y**(*k*,*k*−1) shrinks. The final approximation at column *k* is acceptable if *err*_*k*_≤1, where *err*_*k*_ is a scaled version of *Δ***Y**(*k*,*k*−1) (see Appendix: Stochastic Bulirsch-Stoer full algorithm for more detail). If *err*_*k*_>1, the step is rejected and redone with $\tau =\frac{\tau }{2}$.

In a practical implementation, the initial step tests over *q*=1,…,*k*_*m* *a* *x*_, where *k*_*m* *a* *x*_ is usually set as eight, in order to establish the *k* necessary to achieve the required accuracy and ensure the stepsize is reasonable; subsequent steps then test for convergence only in columns *k*−1,*k* and *k*+1 [28]. Because of its accuracy, the steps taken by the Bulirsch-Stoer method can be relatively large compared to other numerical solvers. *τ* is changed adaptively at each step, and is chosen to minimise the amount of work done (i.e. function evaluations $\hat{n}+1$ of the MMP) per unit stepsize. In this way the Bulirsch-Stoer method adapts its order and stepsize to maximise both accuracy and computational efficiency.

### Stochastic Bulirsch-Stoer method

The SBS method is based on its deterministic counterpart described in the previous section. There are some key issues that must be addressed in order to successfully adapt it into a stochastic method. The two most important ones are interlinked: first, what quantity should be calculated at each step, and second, how can stochasticity be introduced into the picture? The deterministic Bulirsch-Stoer method calculates **X**_*n*+1_ from **X**_*n*_ using the MMP to find the intermediate stages over [*t*,*t*+*τ*). However, stochasticity cannot simply be added to this scheme either inside or outside the MMP, as this would interfere with the extrapolation necessary for the Neville table. In order to update the state vector as in Eq. (1), we must find the number of reactions per step.

Looking at the update formula for the trajectory of a jump Markov process [29],

(5) $$X_{n+1}=X_{n}+\overset{M}{\underset{j=1}{\sum }}\nu_{j}P\left(\overset{t_{n}+\tau }{\underset{t_{n}}{\int }}a_{j}(X(t))\text{dt}\right),$$

it is clear that the quantity we must calculate is $\overset{t_{n}+\tau }{\underset{t_{n}}{\int }}a(X(t))\text{dt}$, in order to then take a Poisson sample for the update (the *τ*-leap method approximates this as *a*(**X**(*t*_*n*_))*τ*). Thus, rather than calculating **X**_*n*+1_ directly using the MMP, we need an accurate way to find the integral of the propensity functions over each step. Proceeding in a somewhat similar way to Algorithm 3, we arrive at Algorithm 4: the intermediate stages are found using the MMP, and the propensities calculated at each stage. These intermediate propensities are then fed into a composite trapezoidal method to give an accurate estimate of the integral.

An important point is that the intermediate stages are solved using the reaction rate equations (Steps 1, 3, 5 of Algorithm 4), which give the expectation of the stochastic trajectory over each step provided both are started in state **X**_*n*_ at time *t*_*n*_. Thus we find the *expected*$\overset{t_{n}+\tau }{\underset{t_{n}}{\int }}a(X(t))\text{dt}$ using Romberg integration, and use this to sample a Poisson distribution in order to increment **X**_*n*_. This method is both extremely accurate at finding the mean and fully stochastic, that is each simulation gives a different stochastic realisation and the full probability density can be found from a histogram of many simulations. 

We have now arrived at the implementation of the SBS. First, we calculate $\Delta a^{{\hat{\tau }}_{q}}(t_{n},t_{n}+\tau )$, the expected integral of the propensities over [*t*_*n*_,*t*_*n*_+*τ*), using Algorithm 4 with multiple stepsizes ${\hat{\tau }}_{1},{\hat{\tau }}_{2},\ldots$. We then extrapolate these using the Neville (Romberg) table to arrive at the extrapolated solutions *Δa*^*e* *x* *t* *r*^(*t*_*n*_,*t*_*n*_+*τ*); this is known as Romberg integration. Once these are sufficiently accurate, we sample the number of reactions as

(6) $$X_{n+1}=X_{n}+\overset{M}{\underset{j=1}{\sum }}\nu_{j}P\left(\Delta a_{j}^{\text{extr}}(t_{n},t_{n}+\tau )\right).$$

This is our approximation to the underlying probability density function at each step. Combined with the extrapolation mechanism described previously and a way to adapt the stepsize, we have the full SBS method.

The stepsize is chosen by calculating the quantity

(7) $$\tau_{k}=\tau \;S_{1}{\left(\frac{S_{2}}{\text{er}r_{k}}\right)}^{\frac{1}{2(k-1)+1}},$$

where *τ* is current timestep, *τ*_*k*_ is the hypothetical next timestep for Romberg table column *k* and *S*_1_ and *S*_2_ are safety parameters, introduced in the next paragraph. Here *err*_*k*_ is the local error relative to a mixed tolerance, with order $O(\tau^{2(k-1)+1})$ (see Appendix: Stochastic Bulirsch-Stoer full algorithm). Its ideal value is exactly one: if it is any smaller than this the step could have been made bigger, and if it is any larger it means our error bound is exceeded and the step must be redone using a smaller *τ*. At each step, a candidate next timestep is selected for each Neville table column *k*. As we know some measure of the work done for each *k* (the number of function evaluations of the MMP), we can calculate the efficiency of each of the *k* candidate timesteps as the work per unit *τ*. We then select the candidate *τ*_*k*_ that gives the highest efficiency. For brevity, we have left the full description and step-by-step implementation of the SBS (Algorithm 5) until the Appendix.

The SBS uses several different parameters (see Algorithm 5), all of which have some effect on the results. *S*_1_ and *S*_2_ are both safety factors that resize the next timestep by some amount: the smaller they are, the smaller the timestep and the more accurate the solution. As always, however, there is a compromise between stepsize and speed, so one must be careful to optimise the parameters for maximum efficiency. The same is also true for the vectors *a*_*t* *o* *l*_, the absolute error tolerance, and *r*_*t* *o* *l*_, the relative error tolerance. These are used to scale the error that is calculated from the internal consistency of the Romberg table. They are usually set fairly low: around 10^−6^ is common. There is an additional consideration with the SBS, namely that of the column of convergence, *k*. Even when the safety factors are set high (meaning larger timesteps), the SBS can achieve very high accuracy by simply doing another extrapolation, and going to a higher column. For this reason, the relationship between the safety factors and accuracy is not a direct one, and it is advisable to check the timesteps and column of convergence for each new set of parameters.

### Extension: SBS-DA

There is an alternative scheme to Eq. (6) for finding the stochastic update to the state vector: this is the ‘degree of advancement’, or DA approach, and we call the resulting method the SBS-DA. Its focus is the *M*×1 random process *Z*_*j*_(*t*),*j*=1,…,*M*, the number of times that each reaction occurs over [0,*t*) [30,31]. *Z*_*j*_(*t*) is related to the state vector **X**(*t*) by

$$X(t)=X(0)+\overset{M}{\underset{j=1}{\sum }}\nu_{j}Z_{j}(t).$$

In fact, **X**(*t*) is uniquely determined by ***Z*** (*t*), where ***Z*** (*t*) is an *M*×1 vector [31]. This allows us to use the DA approach to calculate the number of reactions per step, then return to the population approach to update the state vector, using

(8) $$X(t+\tau )=X(t)+\overset{M}{\underset{j=1}{\sum }}\nu_{j}Z_{j}([t,t+\tau )),$$

where we define *Z*_*j*_([*t*,*t*+*τ*)) as the number of reactions occurring over [*t*,*t*+*τ*). Notice that Eq. (8) has the same form as Eqs. (1) and (6). In fact, *K*_*j*_=*Z*_*j*_([*t*,*t*+*τ*)): in the case of the SSA, the timestep tends to be very small and only one reaction occurs, but for the *τ*-leap and SBS it is much larger so more reactions can occur. Similarly to Eq. (5), we know that [32]

(9) $$K_{j}=P\left(\overset{t+\tau }{\underset{t}{\int }}a_{j}(Z(s))\text{ds}\right).$$

In order to find the update of the state vector, we must solve for the mean (and variance, see below) of *K*_*j*_, and sample according to Eq. (9). The equations for the evolution of the mean and variance of *K*_*j*_,*μ*_*j*_(*s*) and *V*_*j*_(*s*), respectively (where *s* runs only over one step [*t*,*t*+*τ*)), can be derived from its master equation, and take the form [19] (see also [31])

(10) $$\begin{array}{lcr} \frac{d\mu_{j}(s)}{\text{ds}} & = & \overset{M}{\underset{j^{\prime }=1}{\sum }}f_{jj^{\prime }}(X_{n})\mu_{j^{\prime }}(s)+a_{j}(X_{n}),\;\mu_{j}(t)=0, \end{array}$$

(11) $$\begin{array}{lcr} \frac{dV_{j}(s)}{\text{ds}} & = & 2f_{\text{jj}}V_{j}(s)+\frac{d\mu_{j}(s)}{\text{ds}},\;\;\;\;\;V_{j}(t)=0, \end{array}$$

where *s*∈[*t*,*t*+*τ*), **X**_*n*_ is the value of the state vector at the start of the step and $f_{jj^{\prime }}(X_{n})=\overset{N}{\underset{i=1}{\sum }}\frac{\partial a_{j}(X_{n})}{\partial x_{i}}\nu_{ij^{\prime }},j,j^{\prime }=1,\ldots ,M$ are the elements of an *M*×*M* matrix (note that we only deal with its diagonal elements in the case of the variance). Eqs. (10) and (11) must be solved simultaneously with initial conditions *μ*_*j*_(*t*)=*V*_*j*_(*t*)=0 to find *μ*_*j*_(*t*+*τ*) and *V*_*j*_(*t*+*τ*). It should be noted that they are only exact for systems with linear propensities. In the case of non-linear propensities, the moment equations contain higher moments and we obtain Eqs. (10) and (11) by a standard closure argument: we Taylor expand the propensities and truncate at first-order [19]. In fact, Eq. (11) is only necessary because Eq. (10) is not exact in the general case. For larger timesteps this may lead to a sizeable error, so we must approximate the true, Poisson, distribution of *K*_*j*_ with a Gaussian whose variance has been corrected. This leads to the update scheme

$$\;\begin{array}{l} K_{j}(\mu_{j}^{\text{extr}}(t+\tau ),V_{j}^{\text{extr}}(t+\tau ))\\ \;\;=\;\left\{\;\;\begin{array}{c} P(\mu_{j}^{\text{extr}}(t+\tau ))\;\;\;\;\;\;\;\text{if}\;\;\mu_{j}^{\text{extr}}(t+\tau )\;<\;10,\\ \left\lfloor N\left(\mu_{j}^{\text{extr}}(t+\tau ),\sqrt{V_{j}^{\text{extr}}(t+\tau )}\right)\right\rceil \;\text{if}\;\;\mu_{j}^{\text{extr}}(t+\tau )\;\geq \;10, \end{array}\right) \end{array}$$

which now replaces Eq. (6). Here ⌊ ⌉ denote rounding to the nearest integer and the value ten has been chosen heuristically as above this value a Poisson sample can be well represented by a Gaussian sample with the appropriate mean and variance.

This approach is somewhat similar to the unbiased *τ*-leap [19]. The key difference is that Ref.[19] uses this scheme in the context of a fixed-stepsize *τ*-leap method, basing the entire method around this scheme. In contrast, the SBS-DA is grounded in the Bulirsch-Stoer method, which it uses for its stepsize selection and combination of MMP and Richardson extrapolation to find the Poisson increment. The DA approach is only one part of the whole SBS-DA, and is only used as an alternative to Eq. (6), in order to also find the variance of the number of reactions occurring over each step.

The SBS and SBS-DA methods both calculate the parameter of the Poisson sample but they take different approaches to this. The two key differences are that (1) the SBS-DA attempts to correct using the variance of the sampled distribution in order to better approximate the true Poisson parameter when the stepsize is large, but (2) it sacrifices some of its performance because of the inherent inaccuracies of Eqs. (10) and (11) (see Results, Higher order of accuracy and robustness section).

## Results and discussion

To illustrate their effectiveness, we apply the SBS and SBS-DA methods to four example problems of varying degrees of complexity. We compare them with the popular benchmark of the Euler *τ*-leap method (TL; most recent formulation)[14], and we also selected two newer methods that are intended to be representative of the most current, fastest and most accurate methods. These are the *θ*-trapezoidal *τ*-leap (TTTL) [21], which has two stages and weak order two, and the unbiased *τ*-leap (UBTL) [19], which accurately estimates the mean and variance of the number of reactions that occur during one step. Although the authors of these methods have used fixed stepsizes in their works, we have implemented their methods using the same *τ*-adapting scheme as the Euler *τ*-leap. This actually makes them more advanced than originally described, but we believe this ensures a fairer comparison with the SBS.

We use four example problems: a simple chain decay, the Michaelis-Menten reactions, the Schlögl system and the mutually inhibiting enzyme system. All the methods we tested have parameters that can be varied: for the SBS methods these are *r*_*t* *o* *l*_,*a*_*t* *o* *l*_,*S*_1_ and *S*_2_, and for the *τ*-leap methods it is *ε* (and *θ* for the TTTL, which was always set as 0.55). In the former case, we chose to focus our attention on *r*_*t* *o* *l*_, as it plays a somewhat similar role to *ε* in the latter ones, i.e. as a relative bound for the errors. For each system, we produced a plot of the ‘histogram error’ (see below) versus runtime for several values of *r*_*t* *o* *l*_ and *ε*. We only varied these single parameters, listed in Table 2: the other parameters of the SBS were chosen to maximise the overlap between the runtimes of the five methods, and kept constant. This was solely to facilitate comparison between the different methods, and these values do not necessarily fall in the normally useful ranges of those parameters. In order to discriminate between the methods, the plots could be used to choose a CPU time and check which method has the lowest error at that point, or to find which method takes less time to run for a set error level.

**Table 2:** Parameters varied for SBS, SBS-DA, TL, TTTL and UBTL for each test system in order from fastest to slowest (left to right in efficiency plots)

**Figure**	**System**	**Method**	**Parameters (*r***_***tol***_** for SBS methods, *ε* for TL methods)**					
		SBS	10^−5^	5×10^−6^	10^−6^	5×10^−7^	2.5×10^−7^	10^−7^
		SBS-DA	10^−5^	5×10^−6^	10^−6^	5×10^−7^	10^−7^	10^−8^
Figure 2	Chain decay	TL	0.125	0.1	0.075	0.05	0.04	0.03
		TTTL	0.2	0.15	0.1	0.075	0.05	0.04
		UBTL	4	2	1	0.8	0.6	0.5
		SBS	10^−2^	10^−3^	10^−4^	10^−5^	10^−6^	10^−7^
		SBS-DA	10^−3^	10^−4^	10^−5^	10^−6^	10^−7^	10^−8^
Figure 3	Michaelis-Menten	TL	0.2	0.15	0.1	0.07	0.05	0.04
		TTTL	0.3	0.25	0.2	0.1	0.075	0.06
		UBTL	7	5	3	2	1.5	1
		SBS	10^−5^	8×10^−6^	4×10^−6^	10^−6^	5×10^−7^	10^−7^
		SBS-DA	2×10^−4^	1.5×10^−4^	10^−4^	9×10^−5^	8×10^−5^	6×10^−5^
Figure 4	Schlögl	TL	0.046	0.044	0.042	0.04	0.0375	0.035
		TTTL	0.06	0.056	0.052	0.048	0.046	0.044
		UBTL	0.3	0.28	0.26	0.24	0.22	0.2
		SBS	10^−5^	10^−6^	10^−7^	10^−8^	10^−9^	10^−10^
		SBS-DA	10^−5^	10^−6^	10^−7^	10^−8^	10^−9^	10^−10^
Figure 5	Enzymes	TL	0.15	0.1	0.07	0.05	0.03	0.02
		TTTL	0.3	0.2	0.12	0.08	0.06	0.04
		UBTL	5	3	1.5	0.8	0.4	0.3

The same number of simulations were run with all methods. We plotted probability density functions (PDFs) for each species and compared them to ones obtained from a reference set of 10^6^ SSA simulations, all generated from histograms using identical bins. We defined the histogram error as the *L*^1^ distance between the probabilities of each method and the SSA in each bin. The runtime is the time taken to run a single simulation, obtained by dividing the total runtime by the number of simulations.

We show the probability distributions of all the simulation methods, as well as plots of histogram error versus (single) runtime. We refer to the latter as ‘efficiency’ plots, as they clearly indicate some measure of computational efficiency. If a method is both fast and has low error, it is efficient: its points are concentrated towards the origin. In contrast, points to the top right indicate low efficiency (i.e. a slow and inaccurate method).

### Chain decay system

We start with a simple test system that has linear propensity functions (i.e. *a*_*j*_(**x**)∝**x**). The system has three species that are converted into each other by the reactions

$$\begin{array}{lcr} X_{1} & \overset{c_{1}}{\underset{}{\to }} & X_{2},\;c_{1}=1,\\ X_{2} & \overset{c_{2}}{\underset{}{\to }} & X_{3},\;c_{2}=1. \end{array}$$

**Figure 2:**
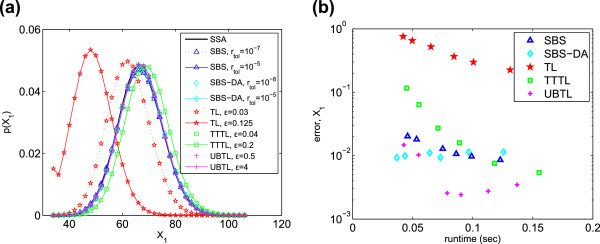
**Chain decay system. (a)** PDFs of *X*_1_ generated from 5×10^5^ simulations. Only the PDFs of the most and least accurate error parameters are shown. **(b)** Histogram error of each method as compared to the PDF of *X*_1_ simulated with the SSA. Parameters varied are listed in Table 2.

The simulations were started in initial state **X**(0)=[10000,1,0]^*T*^ and simulation time was *T*=5. We ran 5×10^5^ simulations. The SBS safety factors were *S*_1_=0.2,*S*_2_=0.4, those for the SBS-DA were *S*_1_=0.15,*S*_2_=0.2, and *a*_*t* *o* *l*_=10^−6^ for both. Probability distributions of the simulation results are shown in Figure 2a for *X*_1_. For clarity, the figure shows only the results for the most and least accurate parameter values. The UBTL and SBS methods’ PDFs both match the SSA very closely; the other methods are less accurate. This is quantified in Figure 2b: the UBTL returns the lowest errors, followed by the SBS-DA and SBS. This is not surprising: for linear systems, the UBTL (and SBS methods) are exact. For this system, taking into account all three chemical species, the SBS methods and the UBTL are the most efficient (Table 3). We have included the efficiency plots for all species in the Additional file, and we have defined a quantity to estimate the total measure of efficiency across all species; these are described in the Further comparisons section.

**Table 3:** **Efficiencies of each method as calculated according to Eq. (**12**) for each test system (higher is better)**

**System**	**SBS**	**SBS-DA**	**TL**	**TTTL**	**UBTL**	**Bin sizes (for species 1,2,…*N*)**
Chain decay	14.7	22.3	0.4	4.4	14.1	2, 5, 5
Michaelis-Menten	7.1	11.7	2.3	7.7	1.2	5, 5, 5, 5
Schlögl	19.1	7.1	18.8	18.8	0.3	10
Enzymes	21.9	50.5	12.6	6.3	3.0	100, 100, 50, 50, 50, 50, 50, 50

Histogram bin sizes for each species are listed in order.

### Michaelis-Menten system

This is a well-known system in biochemistry and is often used to test computational simulations. It is a model of an enzyme (*X*_2_) catalysing the production of some molecule (*X*_4_). It consists of four chemical species undergoing the reactions

$$\begin{array}{lcr} X_{1}+X_{2} & \overset{c_{1}}{\underset{}{\to }} & X_{3},\;\;\;\;\;c_{1}=10^{-4},\\ \;X_{3} & \overset{c_{2}}{\underset{}{\to }} & X_{1}+X_{2},\;c_{2}=0.5,\\ \;X_{3} & \overset{c_{3}}{\underset{}{\to }} & X_{2}+X_{4},\;c_{3}=0.5. \end{array}$$

**Figure 3:**
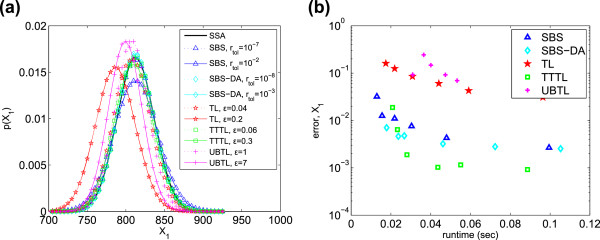
**Michaelis-Menten system. (a)** PDFs of *X*_1_ generated from 10^6^ simulations, and **(b)** histogram error of each method as compared to the PDF of *X*_1_ simulated with the SSA. Parameters varied are listed in Table 2.

The initial state was **X**(0)= [1000,200,2000,0]^*T*^ and the simulation time was *T*=10. We ran 10^6^ simulations. The SBS safety factors were set as *S*_1_=*S*_2_=0.35, and those of the SBS-DA as *S*_1_=*S*_2_=0.33, with *a*_*t* *o* *l*_=10^−6^ for both. The PDFs and efficiency plot for *X*_1_ are shown in Figure 3. The SBS, SBS-DA and TTTL all achieve high accuracy. The TTTL becomes more accurate than the SBS methods at longer runtimes, but the SBS methods have the advantage at shorter runtimes. Thus when it is important to minimise runtime, the SBS methods are preferable. Overall, the SBS-DA has the highest efficiency, with the TTTL second and SBS a close third (Table 3).

### Schlögl system

The Schlögl system is useful as a test system that is both bimodal and non-linear, while at the same time being very simple. It is bimodal in species *X* with a high and a low stable state, although this is only the case for certain parameter combinations. It consists of the four reactions

$$\begin{array}{lll} \;A+2X & \overset{c_{1}}{\to }\;3X, & \;c_{1}=3\times 10^{-7},\\ \;3X & \overset{c_{2}}{\to }\;A+2X, & \;c_{2}=10^{-4},\\ \;B & \overset{c_{3}}{\to }\;X, & \;c_{3}=10^{-3},\\ \;X & \overset{c_{4}}{\to }\;B, & \;c_{4}=3.5, \end{array}$$

and species *A* and *B* are held constant at 10^5^ and 2×10^5^ units respectively. We used the initial condition *X*(0)=250, which is an intermediate value between the two stable states. The simulation time was *T*=10, and we ran 10^5^ simulations for each method. The SBS safety factors were *S*_1_=*S*_2_=0.05, those for the SBS-DA as *S*_1_=*S*_2_=0.125, and *a*_*t* *o* *l*_=10^−6^ for both.

**Figure 4:**
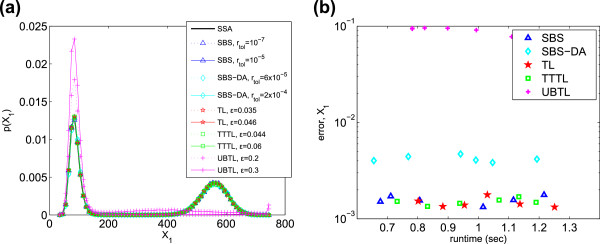
**Schlögl system. (a)** PDFs of *X*_1_ generated from 10^5^ simulations, and **(b)** histogram error of each method as compared to the PDF of *X*_1_ simulated with the SSA. Parameters varied are listed in Table 2.

The PDFs and efficiencies of each method are shown in Figure 4 for *X*_1_. For this system, the TL is surprisingly accurate compared to the other methods. The SBS and TTTL have approximately the same efficiency as the TL, with the SBS-DA being somewhat less efficient and the UBTL the least (Table 3).

### Mutually inhibiting enzymes system

This system has 8 chemical species and 12 reactions [33,34]. It represents the interactions of two enzymes, *E*_*A*_ and *E*_*B*_, and their products, *A* and *B*, respectively. Each enzyme reacts with some substrate (that is not accounted for in the model) to create its product. These products then go on to inhibit the other enzyme. Thus, if initially there are more *E*_*A*_ or *A*, this reduces the chances of *B* being produced, and vice versa. This makes the system bistable in the products. This system is a good example of the double-negative feedback mechanism that is very common in cell biology. Here, however, we use a parameter set that does not result in bistability. The reactions are

$$\begin{array}{lll} \;E_{A} & \overset{c_{1}}{\to }E_{A}+A, & \;c_{1}=15,\\ \;E_{B} & \overset{c_{2}}{\to }E_{B}+B, & \;c_{2}=15,\\ \;E_{A}+B & \overset{c_{3}}{\underset{c_{4}}{⇌}}E_{A}B, & \;c_{3}=5\times 10^{-4},c_{4}=2,\\ E_{A}B+B & \overset{c_{5}}{\underset{c_{6}}{⇌}}E_{A}B_{2}, & \;c_{5}=10^{-3},c_{6}=6,\\ \;A & \overset{c_{7}}{\to }\emptyset , & \;c_{7}=5,\\ \;E_{B}+A & \overset{c_{8}}{\underset{c_{9}}{⇌}}E_{B}A, & \;c_{8}=5\times 10^{-4},c_{9}=2,\\ E_{B}A+A & \overset{c_{10}}{\underset{c_{11}}{⇌}}E_{B}A_{2}, & \;c_{10}=10^{-3},c_{11}=6,\\ \;B & \overset{c_{12}}{\to }\emptyset , & \;c_{12}=5. \end{array}$$

**Figure 5:**
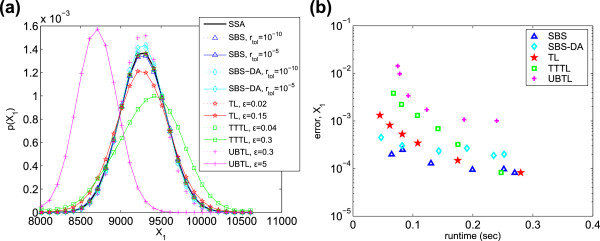
**Mutually inhibiting enzymes system. (a)** PDFs of *X*_1_ generated from 2×10^5^ simulations, **(b)** histogram error of each method as compared to the PDF of *X*_1_ simulated with the SSA. Parameters varied are listed in Table 2.

The initial state was set to **X**(0) = [20000, 15000, 9500, 9500, 2000, 500, 2000, 500]^*T*^, where **X**=[*A*,*B*,*E*_*A*_,*E*_*B*_,*E*_*A*_*B*,*E*_*A*_*B*_2_,*E*_*B*_*A*,*E*_*B*_*A*_2_]^*T*^, and the system was simulated 2×10^5^ times for time *T*=2. We used safety factors of *S*_1_=*S*_2_=0.4 for the SBS and *S*_1_=0.55,*S*_2_=0.7 for the SBS-DA, with *a*_*t* *o* *l*_=10^−6^. The PDFs and efficiencies for *X*_1_ are shown in Figure 5; again the TL is unexpectedly efficient, with only the SBS and SBS-DA more efficient overall (see Table 3). At the longest runtimes, both the TTTL and TL are more accurate than the SBS-DA and similar to the SBS. However, as runtime is decreased, the SBS remains very accurate whilst the TTTL and TL quickly lose accuracy, and for shorter runtimes the SBS-DA is also more accurate than them (Figure 5). Taking into consideration all eight species, it is, in fact, the SBS-DA that is most efficient, followed by the SBS (Table 3).

### Further comparisons

All of our test systems have more than one species, and so far we have only presented results for *X*_1_. This can often be unrepresentative of the full picture. The chain decay system is a clear example of this. Additional file 1: Figure A1 shows the efficiency plots for all three species. Only looking at *X*_1_ could lead one to think that the UBTL is the most efficient method for simulating this system. But including the other two species reveals that the SBS-DA is, in fact, the most efficient overall. This is important, because it is clear that factors such as linear/non-linear propensities, population size and stiffness all affect each reaction and species in a different way. Thus it is *overall* performance we are interested in.

To overcome this problem, we use a way of quantifying the overall efficiency of each method over all species. This follows directly on from our previous definition of efficiency: low error and low runtime implies an efficient method, high error and high runtime implies an inefficient method, and a combination of the two, for instance high error but low runtime, clearly lies somewhere between the two. We define ‘efficiency’ *η* as

(12) $$\;\eta \;=\;\frac{{\text{(sum(total error over all histogram bins and all species))}}^{-1}}{\text{sum(single-simulation runtime over all error parameters)}}\;.$$

This varies for each system, and is comparable *only* when the bins used to calculate errors are identical. In other words, the values are a direct comparison of the efficiency of each method for each test system, but should not be used across different test systems. Table 3 compares the efficiencies of each simulation method for every test system.

Additional file 1: Figures A1 to A4 contain a full picture of our computational results for all four example systems and all simulation methods. The overarching trend was the following: the SBS was very accurate, returning the lowest error in many cases. The TL was unexpectedly efficient for some systems. The TTTL also achieved good efficiency for longer runtimes, but the SBS had a flatter efficiency curve than the TTTL, with the TTTL quickly losing accuracy at lower runtimes even when it was more accurate than the SBS at higher runtimes. A clear trend emerges: the SBS is a very accurate method. Moreover, it is the most *efficient* method we tested, maintaining its accuracy better at low runtimes than the other methods.

SBS methods excel when we want a short runtime with high accuracy. In this case, we set the safety factors high, allowing large steps and a corresponding increase in extrapolations. This retains a high accuracy, whilst reducing runtime because of the large timesteps. In contrast, when we allow a longer runtime, we set the safety factors low, restricting the stepsize and removing the need for higher extrapolation. In many of our test examples, we have seen the SBS only using one extrapolation throughout the simulation. This is a waste of the extrapolation capability of the SBS, and it is no surprise that in these cases it is not the most efficient method, especially as the stepsize adaptation scheme adds some overhead to each step.

### Higher order of accuracy and robustness

A thorough study of the order properties of *τ*-leaping methods was first given by Rathinam *et al.*[35], who showed that for linear reactions the Euler *τ*-leap method is weak order one in the moments under the scaling *τ*→0. This analysis was extended by Li [36] to non-linear propensity functions by considering SDEs driven by Poisson random measures (see also [37]). Li showed that the Euler *τ*-leap method is precisely the Euler method applied to this SDE and hence inherits the properties of strong order half and weak order one. However, there are issues with using the scaling condition *τ*→0 as the *τ*-leap condition requires that $\sum a_{j}(X)\tau \gg 1$. Anderson *et al.*[38] overcame this scaling condition by considering order under a large volume scaling *Ω*→*∞*. In this case, by letting X/Ω=O(1) and with *τ*=*Ω*^−*β*^,0<*β*<1, global strong and weak order convergence can be established. Hu *et al.*[39] investigate these issues in greater detail through the use of rooted tree expansions of the local truncation errors for the moments and covariance, thus generalising the approach first applied to SDEs by Burrage and Burrage [40]. This analysis shows that while some *τ*-leap methods may have higher order moments (for instance, the midpoint *τ*-leap has order two moments for linear systems), their covariance is invariably of unit order, unless this is specially taken into consideration (as with the TTTL method, which has order two moments and covariance). As Hu *et al.*[39] point out, these issues arise as a consequence of the differences between the infinitesimal generators for deterministic ODEs and jump processes.

It is well-known that the Bulirsch-Stoer method has a high order of accuracy: this is the reason it is able to use large steps whilst still finding very accurate solutions. This is because of the Richardson extrapolation that is used at each step on the MMP solutions (which themselves have order two as well as an error expansion containing only even powers of $\hat{\tau }$, resulting in very high order solutions with little work). In contrast, rather than the MMP solutions for **X**(*t*), the SBS instead extrapolates at each step the deterministic quantities $\Delta a^{{\hat{\tau }}_{q}}(t_{n},t_{n}+\tau )$ (or the mean and variance of *K*_*j*_ given by Eqs. (10) and (11) in the case of the SBS-DA), calculated using the composite trapezoidal rule, which also has a known error expansion. Thus the extrapolation is performed on a deterministic variable: the mean of the Poisson update.

We investigated the behaviour of the SBS and SBS-DA methods on two simple systems: first, the linear system *X*→2*X*, *X*(0)=1000, and second, the non-linear system *X*+*Y*→*∅*, *X*(0)=*Y*(0)=10000. As the SBS changes both stepsize and Romberg table column *k* (i.e. order of accuracy) adaptively, we used a restricted version, which had both a fixed stepsize and *k*. We ran simulations with both SBS and SBS-DA, for *k*=1 and *k*=2, that is no extrapolation and one extrapolation, respectively. In addition, we also used the TL and TTTL methods for comparison, as they have known weak orders of accuracy (one and two, respectively). The gradients of the errors for the different methods are computed based on a linear least-squares regression of the data points.

**Figure 6:**
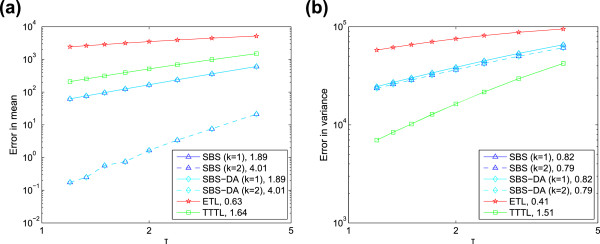
**Order of accuracy for linear system.** Error versus stepsize for **(a)** mean, and **(b)** variance of the linear system *X*→2*X*, with *c*=0.2, *X*(0)=1000, *T*=12. The gradients of linear regression lines fitted to the points are shown in the legend.

**Figure 7:**
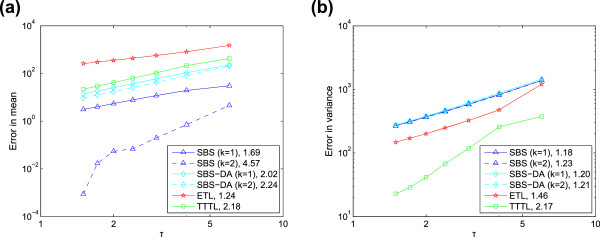
**Order of accuracy for non-linear system.** Error versus stepsize for **(a)** mean, and **(b)** variance of the non-linear system *X*+*Y*→*∅*, with *c*=10^−5^, *X*(0)=[10000,10000]^*T*^,*T*=12. The gradients of linear regression lines fitted to the points are shown in the legend. The first three points of the SBS with *k*=2 are omitted from the regression line, as they are clearly affected by Monte Carlo error.

We find that both SBS and SBS-DA can have high weak order in the mean (in certain cases, such as for large timesteps and populations; this is discussed below). For the linear system, both methods have weak order approximately two and four in the mean for *k*=1 and *k*=2, respectively (Figure 6). However, there is a difference in behaviour between the SBS and SBS-DA for the non-linear system (Figure 7). Here, the SBS-DA is limited to at most weak order two in the mean, even when extrapolated. In contrast, the SBS is limited to at most weak order four when extrapolated. This shows the limitations of Eqs. (10) and (11): for non-linear systems, they limit the order of accuracy of the mean of the SBS-DA to two. This is not the case for linear systems, as here Eq. (10) is exact, so the order of the SBS and SBS-DA is identical.The clear message we can take from Figures 6 and 7 is that the SBS does behave as if it had higher weak order in the moments, and this order increases as the Romberg table column (that is, number of extrapolations) is increased. However, we cannot tell whether this trend continues to higher extrapolations as these are so accurate that Monte Carlo error interferes with our ability to reveal the weak order. On the other hand, the SBS-DA behaves as if it has at most weak order two in the mean for non-linear systems, but this restriction in weak order is compensated for by the use of an appropriate Gaussian sample when the Poisson parameter is large, and generally it is similarly, or even more, efficient than the SBS. However, in neither case does extrapolation increase the weak order of the variance beyond one.Thus one can legitimately ask whether our approach offers any advantage over, for example, the TTTL method, which has weak order two in both the moments and the variance. This can be addressed by perusal of Figures 2, 3, 4 and 5, where we compare the distances of the PDFs of the numerical methods and the exact solution (as computed by the SSA) as a function of the runtime. Of the methods tested the SBS appears to be the most robust and efficient, even though the TTTL has weak order two in the variance. It is the criteria of efficiency and robustness that are the most important properties of any good numerical method. We claim that these properties are intrinsic to the SBS along with its ability to adaptively select the timestep and number of extrapolations to carry out, thus maximising efficiency whilst keeping accuracy high.

Now we discuss the order of accuracy behaviour of our methods. First of all, we are not claiming that they have high weak order uniformly in the stepsize, and we have no such proof apart from in the linear case when the higher order is inherited directly from the underlying deterministic extrapolation methods. However, this statement gives us a key insight into considering the behaviour of numerical methods when applied to SDEs with small noise of the form

(13) $$dX(t)=f(t,X(t))\text{dt}+\mathrm{\varepsilon g}(t,X(t))\text{dW}(t),\;X(t_{0})=x_{0},$$

where *ε*>0 is a small-noise term. It is well-known that Langevin SDEs represent an intermediate regime between discrete stochastic chemical kinetics and the deterministic regime, arising as the number of molecules *X* in the system increases. In particular, *ε* behaves as $\frac{1}{\sqrt{X}}$[11]. For such systems, Milstein and Tretyakov [42] showed that the global weak order of numerical methods to solve the above SDE has the general form $O(\tau^{p}+\tau^{q}\varepsilon^{r})$, where *q*<*p*. When noise is ignored (*ε*=0), the SDE becomes an ODE and the weak order of its approximate solution is just the deterministic order term $O(\tau^{p})$. Milstein and Tretyakov [41] also performed an analysis in the strong sense, and again found the general form of the global strong error to be $O(\tau^{p}+\tau^{q}\varepsilon^{r}),q<p$. In addition, Buckwar *et al.*[43] also examined small-noise SDEs in a strong sense for some well-known classes of Runge-Kutta methods. The implication of the extra term in the stochastic order is that although the underlying deterministic order of the method may be high, the stochastic order is restricted by the noise term. However, when the noise is small, this term will also become small, thus allowing the stochastic order to increase, possibly even up to the deterministic order $O(\tau^{p})$. This is also the case if the stepsize is large. In fact, it occurs over a range of values of *τ* and *ε*: it is trivial to see that the condition for the deterministic term to dominate is $\tau \gg \varepsilon^{\frac{r}{p-q}}$.

Now, a standard way of mathematically investigating discrete stochastic methods is to analyse SDEs with jumps; this is the approach of Li [36]. In particular, Li [36] shows that the Euler discretisation of such an SDE is the Euler *τ*-leap method. Our hypothesis is that the analysis of Milstein and Tretyakov [42] is also applicable to SDEs with jumps; this then tells us something about the behaviour of discrete stochastic methods when noise levels are medium or small. A small-noise analysis similar to that of Milstein and Tretyakov [42] for the SBS is beyond the scope of this paper but we postulate that there is a small-noise error expansion in both *τ* and *ε* for the SBS method. The SBS is most useful when applied to systems with relatively larger biochemical populations (thus small noise), as it is in these cases that the SSA is prohibitively slow and an approximate method is necessary. Combined with the fact that the SBS often uses large stepsizes, this implies that in many systems of interest, the global weak order of the SBS may, in fact, not be far from its deterministic (high) order. This also explains the behaviour of the SBS in our numerical tests, where the timesteps were large and the populations moderate (implying moderate noise), thus making it likely that the condition for the deterministic order term to dominate was met.

### Implementation issues

**Figure 8:**
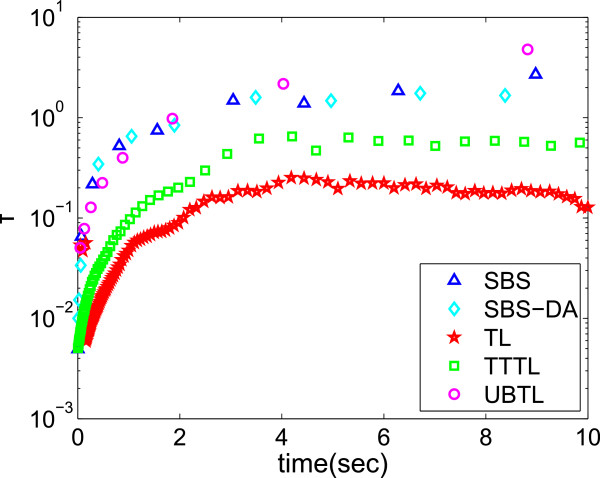
**Stepsizes over time.** Evolution in time of stepsizes of all the stochastic solvers we have compared in the case of the Michaelis-Menten system. The largest steps are taken by the SBS, SBS-DA and unbiased *τ*-leap, then the *θ*-trapezoidal *τ*-leap, and finally the Euler *τ*-leap. Parameters used were: *S*_1_=*S*_2_=0.8,*a*_*tol*_=10^−6^,*r*_*tol*_=10^−4^ for the SBS methods, and *ε*=0.04,0.1,1 for the Euler, *θ*-trapezoidal and unbiased *τ*-leap methods, respectively.

The speed of the SBS is due to the large steps it takes compared to other solvers. We compare the stepsizes for all five methods we used in this paper on the Michaelis-Menten system (Figure 8). Clearly, the stepsizes are influenced by our choice of error parameters. We controlled for this by using parameters that gave similar (or as close as possible) error levels, regardless of runtime. Figure 8 shows that the largest steps were taken by the SBS methods and the UBTL, with the stepsizes being very similar, followed by the other two methods. This is not very surprising: the UBTL is in some respects similar to the SBS-DA, in that it also finds very accurate solutions for the moments of *K*_*j*_ at each step. However, the stepsize is controlled using a completely different mechanism, so it is interesting to see that both employ a similar stepsize for a similar error level in this case.

**Figure 9:**
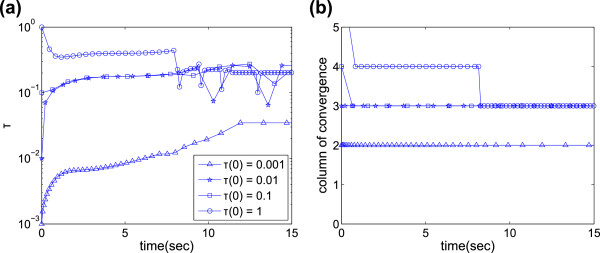
**SBS regimes for chain decay example.** Evolution in time of **(a)** stepsize using different initial stepsizes *τ*(0), and **(b)** the column of the Romberg table at which the solutions converge sufficiently (this is not necessarily *k*, as the error level could be accepted at only *k*−1 or even *k*+1). SBS parameters are *S*_1_=*S*_2_=0.5,*a*_*tol*_=*r*_*tol*_=10^−6^. For clarity not every point has been given a marker.

One peculiarity of the SBS is that it can settle into one of several different ‘regimes’: because it builds the Romberg table adaptively, it can achieve the same accuracy using a larger step and higher extrapolation (i.e. higher Romberg table column) or smaller step and lower extrapolation. The regime into which the particular simulation falls is strongly influenced by the initial stepsize *τ*(0), but often changes mid-simulation. For instance, a smaller *τ*(0) is more likely to fall into the smaller- *τ* (and lower extrapolation) regime, and vice versa. Figure 9 shows how *τ* changes with time in chain decay system simulations using several different *τ*(0). It is clear that there are two regimes, one high- *τ* and one low- *τ*. When *τ*(0) is very small, *τ* settles down to the low regime, and only the second Romberg table column is used; as *τ*(0) is increased, *τ* settles in the high regime and uses the third column. When *τ*(0)=1, *τ* initially enters an even higher- *τ* regime using the fourth column, but eventually settles into the high regime with the third column. In practice, it is advisable to bear this in mind, and choose *τ*(0) accordingly: low- *τ*, low-column simulations are more computationally expensive and if the same accuracy can be achieved with a larger timestep then efficiency can be improved even further.

There are two distinct approaches to determining *τ*(0): first, as described previously, we could set *τ*(0) to an arbitrary value and run the initial step through as many columns as necessary (up to *k*_*m* *a* *x*_) until it finds the required accuracy. Should *τ*(0) be so large that it drives the populations negative, it would also be reduced here until it reaches a more suitable size for the given problem. In addition, if *τ*(0) is still larger than its optimum value, it is reduced over the next several steps until it has reached this optimum value (and vice versa if it is too small). This is the standard approach for the deterministic Bulirsch-Stoer method, and it is the one we have taken in our simulations. However, in the stochastic regime there is another approach: we could set *τ*(0) as some multiple of 1/*a*_0_(**x**_0_) (the expected size of an SSA step in state **x**_0_[12]), along with an initial guess of the Romberg table column to aim for. As 1/*a*_0_(**x**_0_) is very small, this is a more conservative approach, but *τ* is increased to its optimum value over the first few steps. It could be useful for systems that are very stiff, or that oscillate, whose timestep must be very small at certain parts of the solution domain and larger timesteps could result in large errors. There seems to be no substantial difference in accuracy between the two approaches, and we believe both are equally valid.

## Conclusions

Our results have shown that the SBS is generally a very accurate method, at least comparable to or, in most cases, better than its competitors. However, the real strength of the SBS is this accuracy *combined* with the fact that its efficiency curve has a relatively low gradient; in other words, it is an accurate method that loses little of its accuracy as it is speeded up, allowing for fast, robust and accurate simulations. This is because as runtime is shortened, the SBS uses more and more extrapolations to maintain its accuracy. At the same time, the use of larger timesteps means less overhead overall, allowing the SBS to be very efficient. It is in such parameter regimes that the SBS can achieve its full potential. In addition to this, we believe the SBS is also able to achieve high weak order (in the moments; the variance remains one) in the small-to-moderate noise regime, that is when the number of molecules in the system is moderate to large, and also when the timesteps are large compared to the noise level. Its performance in this regime is accelerated as more and more extrapolations are performed, giving it exceptional accuracy.

As the SBS is an explicit method, it is not necessarily suited for solving especially stiff problems. In such cases, Runge-Kutta methods with larger regions of stability, such as the stochastic Runge-Kutta method [44], are more ideal, as well as implicit or multiscale methods [45-47]. The initial stepsize of the SBS should be chosen appropriately, as it may be possible for the SBS to settle in a higher-stepsize regime, which could affect accuracy, or a low-stepsize regime, which could affect runtime. In addition, *τ*(0) should be chosen such that it is within the stability region of the modified midpoint method. Running a few preliminary simulations can help choose *τ*(0).

In previous work, we have extended Richardson extrapolation into the discrete stochastic regime [22]. In this framework, full simulations with fixed stepsize are run over *t*=[0,*T*], and their *moments* are extrapolated to find accurate approximations to the moments at time *T*. In contrast, the SBS uses extrapolation *within* each timestep and varies *τ* to optimise efficiency. Thus the SBS is a complementary approach to extrapolated *τ*-leap methods that has two advantages: first, the stepsize can be adapted to lower runtime and eliminate the need for finding a suitable range of fixed stepsizes; second, the SBS returns an entire histogram, rather than just the moments. This can be desirable in many cases, especially if the solutions do not follow a simple distribution such as a Gaussian or Poisson, or have multiple stable states.

In this paper we have introduced a new efficient and robust simulation method, the Stochastic Bulirsch-Stoer method, which can also achieve higher weak order in the moments for certain systems. This is inspired by the deterministic method of the same name, and as such it also boasts the two main advantages of that method: its speed and its high accuracy. We have shown using numerical simulations that for a range of example problems, it is generally the most efficient and robust out of all recent *τ*-leap methods that we tested, which are the current state-of-the-art in fast stochastic simulation. Thus the SBS is a promising new method to address the need for fast and efficient discrete stochastic methods.

## Appendix: Stochastic Bulirsch-Stoer full algorithm

Here we explain in detail the Stochastic Bulirsch-Stoer method. The aim of the stepsize adapting mechanism of the SBS (shared with the deterministic Bulirsch-Stoer method) is to select the optimal column *k* of the Romberg table (Table 1) that will give an acceptably low error while requiring as little computational work as possible. We define the error of each Romberg column *q* as

(14) $$\text{er}r_{q}=\left|\frac{\mu_{\tau }^{{\hat{\tau }}_{1}{\hat{\tau }}_{q}}-\mu_{\tau }^{{\hat{\tau }}_{2}{\hat{\tau }}_{q}}}{a_{\text{tol}}+r_{\text{tol}}\times \mu_{\tau }^{{\hat{\tau }}_{1}{\hat{\tau }}_{q}}}\right|,$$

where *μ*_*τ*_≡*μ*_*j*_(*τ*) is the mean of *K*_*j*_ as defined in Eq. (10), which is equivalent to $\Delta a^{\hat{\tau }}(t_{n},t_{n}+\tau )$ from Algorithm 4, and | ***v*** | denotes the *L*^2^ norm of the vector ***v***. The most ideal situation is if the error of the *k*-th column, *err*_*k*_=1: if it is larger than one, accuracy has been lost because *τ* was too large; if it is smaller than one, computational time has been lost because *τ* was unnecessarily small. Below, we follow Refs. [24,28] in our exposition. An idea of how *τ* can be adjusted to its optimal value for the next step is given by

$$\tau_{q}=\tau \;S_{1}{\left(\frac{S_{2}}{\text{er}r_{q}}\right)}^{\frac{1}{2(q-1)+1}},\;q=1,\ldots ,k,$$

where *τ*_*q*_ is a set of hypothetical new stepsizes adjusted from the current stepsize *τ*. *S*_1_ and *S*_2_ are safety factors 0<*S*_1_,*S*_2_<1, that ensure *τ* is not set too large because of errors in the MMP and composite trapezoidal rule approximations.

We want the column that minimises the work done per unit step. This is defined for column *q* as

$$W_{q}=\frac{A_{q}}{\tau_{q}},\;q=1,2,\ldots ,$$

where *A*_*q*_ is the work done in computing the *q*-th Romberg table row and is assumed to be the number of function evaluations inside the MMP. An MMP with stepsize $\hat{\tau }=\tau /2$ needs three evaluations, i.e. *A*_1_=3 using our scheme; this can be generalised to

$$A_{q+1}=A_{q}+n_{q+1},$$

where *n*_*q*_=2*q*. The optimal column *k* for the next timestep is given by the lowest *W*_*q*_, and the optimal stepsize by the corresponding *τ*_*q*_. In reality, after the initial step only columns *k*−1, *k* and *k*+1 are tested for convergence, as otherwise the convergence is likely to be an artifact or the timestep is far off its optimal size. This helps reduce the runtime but makes the implementation more complicated.

Now that the reasoning behind the adaptive mechanism is clear, we set out a detailed algorithm for a practical implementation of the Stochastic Bulirsch-Stoer method. To implement the SBS-DA instead, Algorithm 4 should be replaced with Algorithm 3, which would calculate the mean and variance of *K*_*j*_ according to Eqs. (10) and (11). In addition, there should be two Neville tables, one for the mean and one for the variance, which find the extrapolated solutions to each. 

## Abbreviations

SSA: Stochastic simulation algorithm; SBS: Stochastic Bulirsch-Stoer; ODE: Ordinary differential equation; MMP: Modified midpoint method; DA: Degree of advancement; PDF: Probability density function; TL: (Euler) *τ*-leap; TTTL: *θ*-trapezoidal *τ*-leap; UBTL: Unbiased *τ*-leap.

## Competing interests

The authors declare that they have no competing interests.

## Authors’ contributions

MB conceived the SBS idea and supervised the work. TSz and MB jointly developed the method and analysed the results. TSz implemented the method, performed the simulations and drafted the manuscript. KB and KCZ helped with the analysis and provided theoretical insight. All authors took part in revising the manuscript, and all authors have read and approved the final manuscript.

## Supplementary Material

Additional file 1 **Supplementary information.** These show full sets of simulation results for all chemical species of all four test systems, using all the simulation methods we tested. Click here for file
