# Supplementary material for: Efficient simulation of stochastic chemical kinetics with the Stochastic Bulirsch-Stoer extrapolation method
Source: BMC Syst Biol. 2014 Jun 18;8:71. doi: 10.1186/1752-0509-8-71 (PMC4085235; doi:10.1186/1752-0509-8-71)
Supplement: Additional file 1 — Supplementary information. These show full sets of simulation results for all chemical species of all four test systems, using all the simulation methods we tested. [file 1752-0509-8-71-S1.pdf]

Efficient simulation of stochastic chemical kinetics with the Stochastic Bulirsch-Stoer extrapolation method: Supplementary information

Tamás Székely Jr.*[1] , Kevin Burrage[1,2] , Konstantinos C. Zygalakis[3] , Manuel Barrio*[4]

[1]Department of Computer Science, University of Oxford, Oxford, OX1 3QD, United Kingdom
[2]Department of Mathematics, Queensland University of Technology, Brisbane, Qld 4001, Australia
[3]Mathematics, University of Southampton, Southampton, SO17 1BJ, United Kingdom
[4]Departamento de Informática, Universidad de Valladolid, 47011 Valladolid, Spain

Email: Tamás Székely Jr.*- tamas.szekely@cs.ox.ac.uk; Kevin Burrage - kevin.burrage@cs.ox.ac.uk; Konstantinos C. Zygalakis - k.zygalakis@soton.ac.uk; Manuel Barrio*- mbarrio@infor.uva.es;

*Corresponding author

In this file we show the plots for error versus runtime for all the species of the chain decay, Michaelis-Menten, Schlögl and mutually inhibiting enzymes systems.

Chain decay system

Fig. A1 shows histogram errors for all species from 5×10^5 SBS, SBS-DA, TL, TTTL and UBTL simulations. The SBS safety factors are $S_1 = 0.2, S_2 = 0.4$, those for the SBS-DA are $S_1 = 0.15, S_2 = 0.2, a_{tol} = 10^{-6}$, and error parameters corresponding to each point (going left to right) are listed in Table 3 of the article. TTTL parameter $\theta = 0.55$.

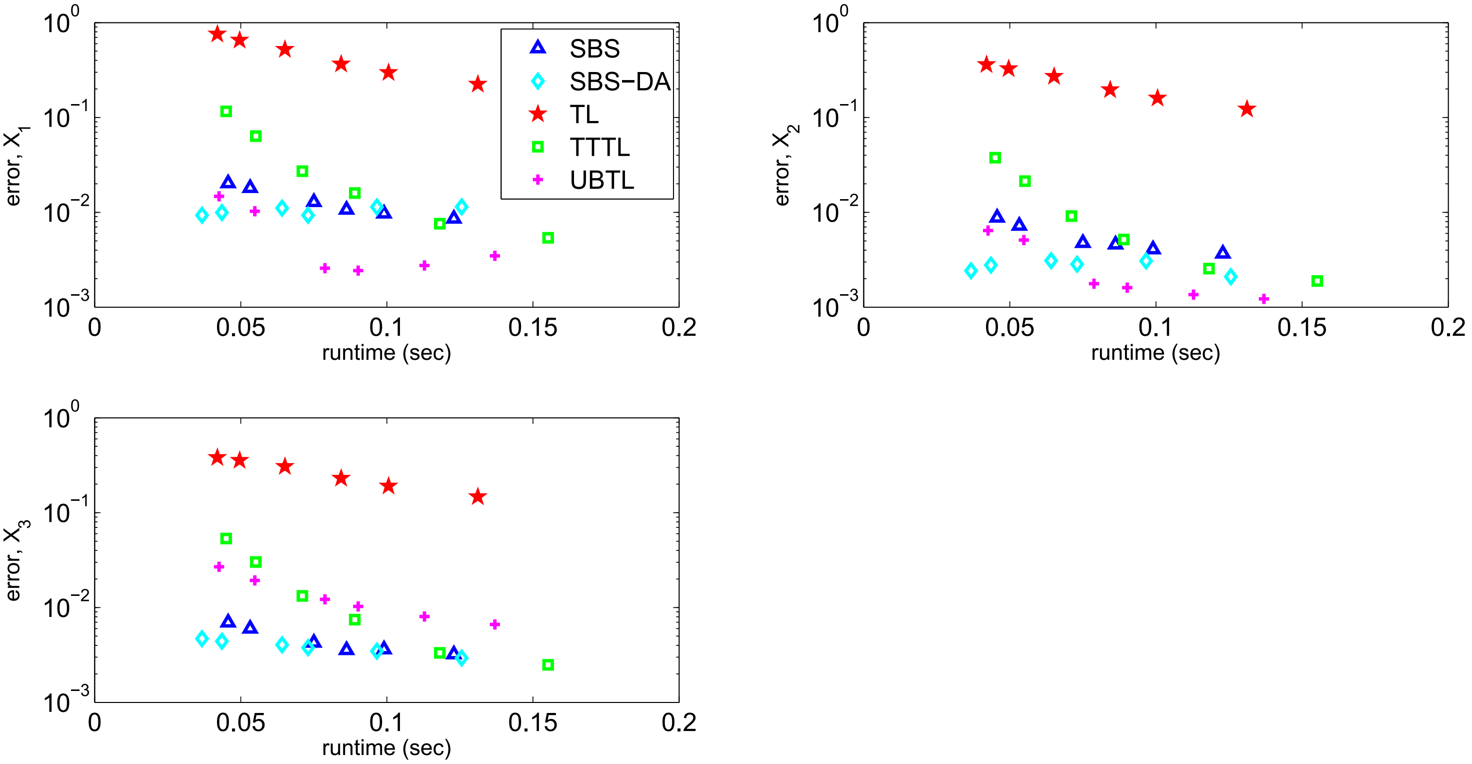

Figure A1: **Chain decay system efficiency for all species and all methods.** Histogram errors of all three species versus time for a single simulation.

Michaelis-Menten system

Fig. A2 shows histogram errors for all species from 10^6 SBS, SBS-DA, TL, TTTL and UBTL simulations. The SBS safety factors are $S_1 = S_2 = 0.35$, and those of the SBS-DA are $S_1 = S_2 = 0.33, a_{tol} = 10^{-6}$, and error parameters corresponding to each point (going left to right) are listed in Table 3 of the article. TTTL parameter $\theta = 0.55$.

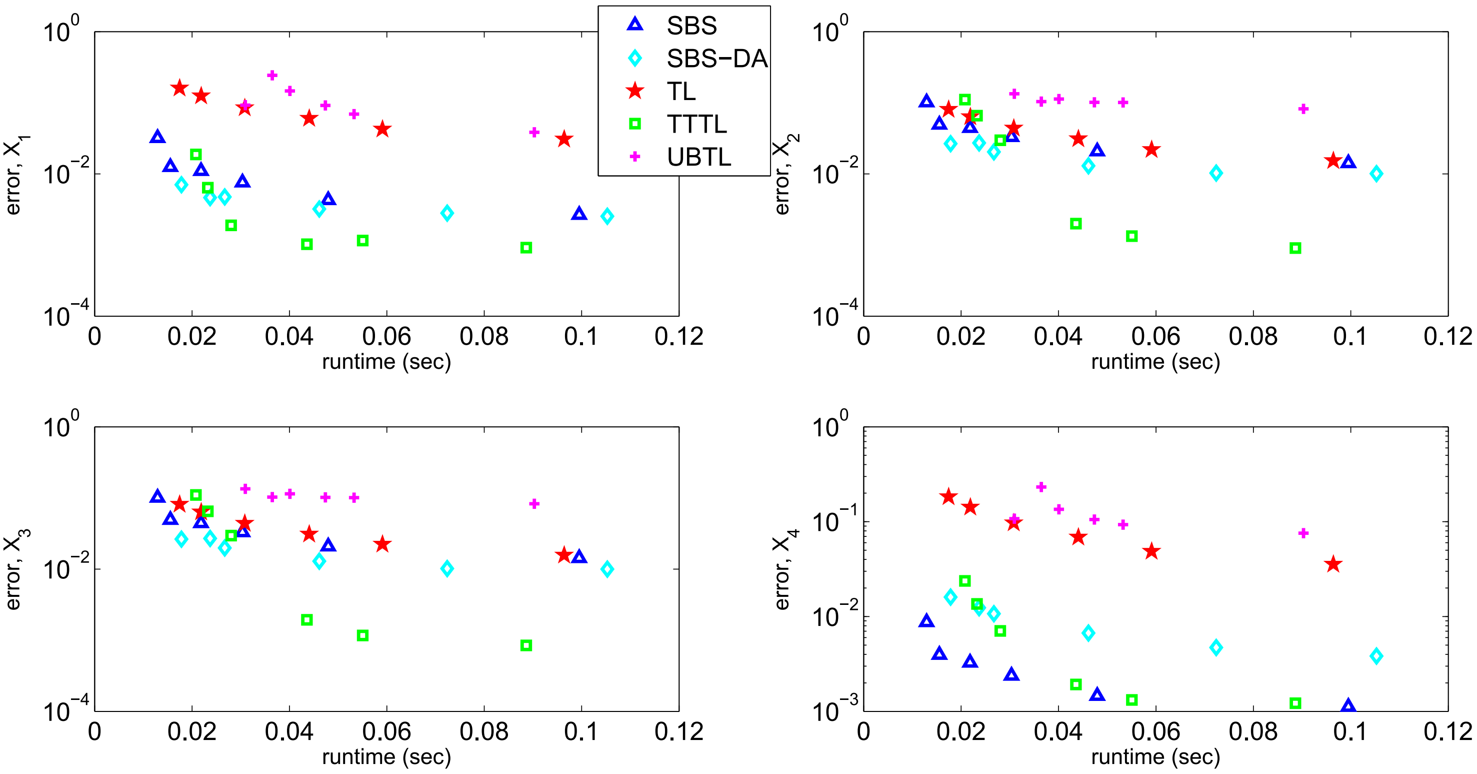

Figure A2: **Michaelis-Menten system efficiency for all species and all methods.** Histogram errors of all four species versus time for a single simulation.

Schlögl system

Fig. A3 shows histogram errors for all species from 2×10^5 SBS, SBS-DA, TL, TTTL and UBTL simulations. The SBS safety factors are $S_1 = S_2 = 0.05$, those for the SBS-DA are $S_1 = S_2 = 0.125, a_{tol} = 10^{-6}$, and error parameters corresponding to each point (going left to right) are listed in Table 3 of the article. TTTL parameter $\theta = 0.55$.

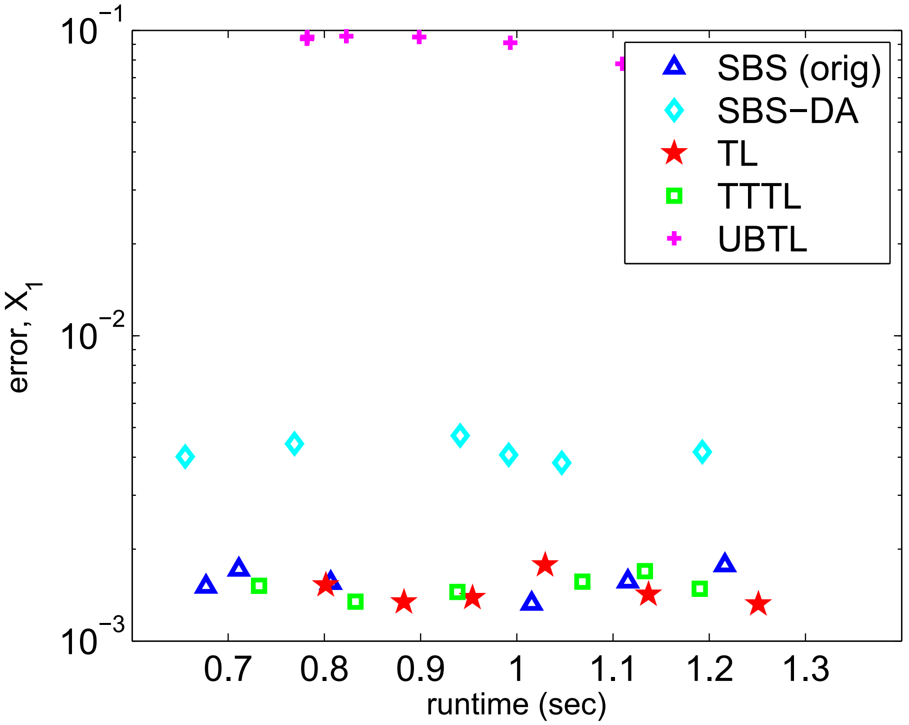

Figure A3: **Schlögl system efficiency for the only species and all methods.** Histogram errors of all four species versus time for a single simulation.

Mutually inhibiting enzymes system

Fig. A4 shows histogram errors for all species from 2×10^5 SBS, TL, TTTL and UBTL simulations. The SBS safety factors are $S_1 = S_2 = 0.4$ and $S_1 = 0.55, S_2 = 0.7$ for the SBS-DA, with $a_{tol} = 10^{-6}$, and error parameters corresponding to each point (going left to right) are listed in Table 3 of the main text. TTTL parameter $\theta = 0.55$.

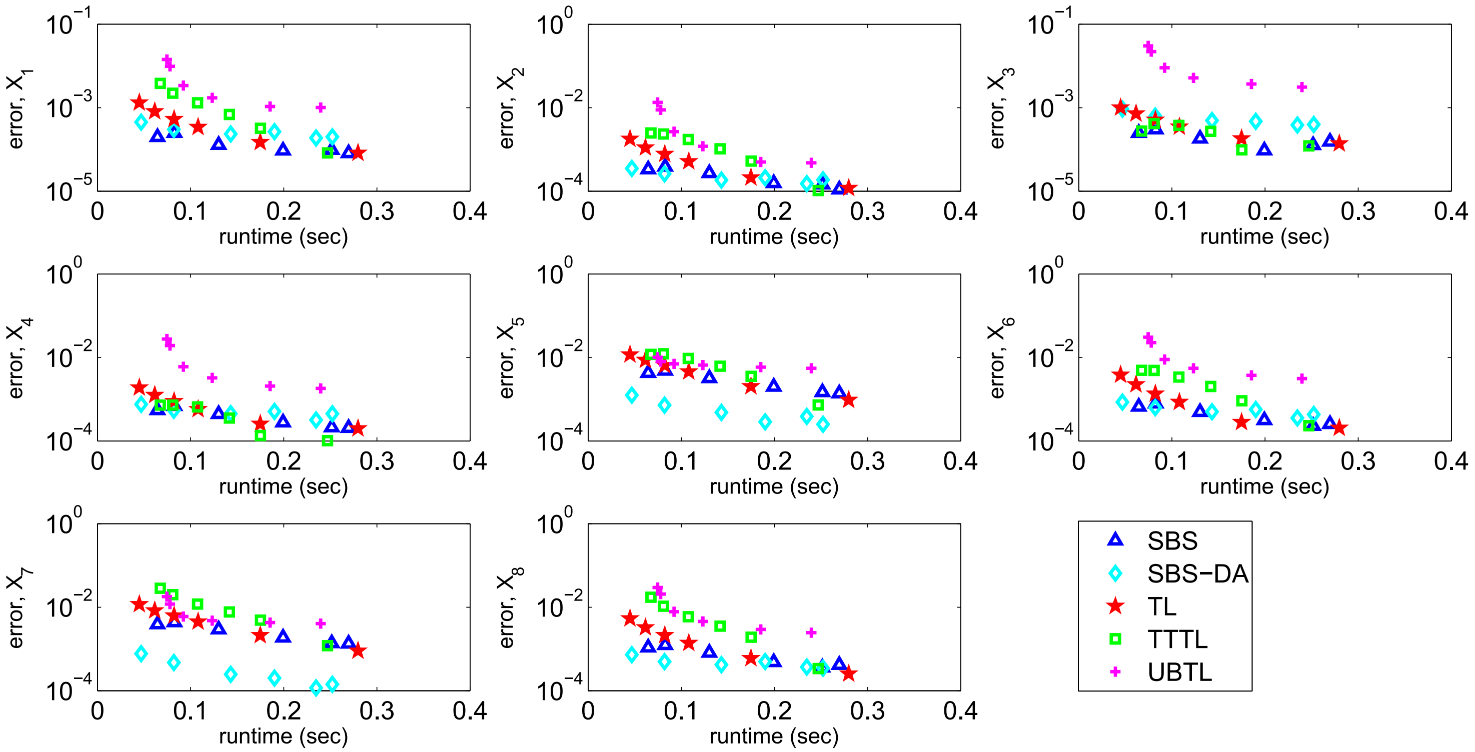

Figure A4: **Mutually inhibiting enzymes system efficiency for all species and all methods.** Histogram errors of all eight species versus time for a single simulation.
